# Comparative outcomes between all-inside arthroscopic suture anchor technique versus arthroscopic transosseous suture technique in patients with triangular fibrocartilage complex tear: a retrospective comparative study

**DOI:** 10.1186/s13018-021-02752-4

**Published:** 2021-10-14

**Authors:** Chia-Hung Hung, Yu-Feng Kuo, Yu-Jen Chen, Ping-Chun Yeh, Hsiao-Yun Cho, Yeong-Jang Chen

**Affiliations:** 1 Department of Orthopedics, Fu Jen Catholic University Hospital, Fu Jen Catholic University , New Taipei City, 24352 Taiwan, ROC; 2 School of Medicine, College of Medicine, Fu Jen Catholic University, New Taipei, 24205 Taiwan, ROC; 3 Graduate Institute of Business Administration, College of Management, Fu Jen Catholic University, New Taipei City, 24205 Taiwan, ROC; 4 Research and Development Center for Physical Education, Health, and Information Technology, Fu Jen Catholic University, New Taipei City, 24205 Taiwan, ROC; 5 Department of Otorhinolaryngology, Head of Neck and Surgery, Fu Jen Catholic University Hospital, Fu Jen Catholic University, New Taipei City, 24352 Taiwan, ROC

## Abstract

**Background:**

Triangular fibrocartilage complex (TFCC) has become an interest over the last few decades, discovering its understanding in anatomy, pathomechanism, biomechanics, and management in treatments. Currently, TFCC does not have a golden standard procedure, and not one surgical procedure is superior to the other. This study is to evaluate the comparative outcomes in TFCC patients that underwent either in all-inside arthroscopic suture anchors or the arthroscopic transosseous suture technique.

**Method:**

From 2017 to 2019, 30 patients were analyzed. Eight patients were in an arthroscopic transosseous group and 22 patients were in an all-inside arthroscopic group. Comparison between patients’ flexion and extension range of motion (ROM), grip strength, and visual analog pain scale (VAS) preoperative and six-month follow-up were analyzed.

**Result:**

There were significant increases in flexion ROM, extension ROM, and VAS between preoperative and postoperative in all-inside arthroscopic and arthroscopic transosseous. Only the all-inside arthroscopic group had a significant increase in grip strength. Postoperative flexion ROM had a significant difference between all-inside arthroscopic and arthroscopic transosseous.

**Conclusion:**

Both the all-inside arthroscopic suture anchor technique and the arthroscopic transosseous suture technique are appropriate treatments to treat patients with TFCC. Both procedures have achieved the ultimate goal of improved longevity and optimal function.

**Level of evidence:**

Level III; retrospective comparative cohort study.

## Introduction

Triangular fibrocartilage complex (TFCC) is one of the major causes of ulnar-sided wrist pain [1]. It consists of soft tissues extending from the distal radius, ulnar, fovea, and to the base of the ulnar styloid. TFCC is not only an important stabilizer of the distal radioulnar joint (DRUJ) but is also a stabilizer to the ulnocarpal joint and distributer between ulnar and ulnar carpus loading [2]. When symptoms of wrist pain are shown, a decrease in grip strength and impaired hand function with an increase in pain will occur. The initial conservative management is commonly treated by immobilization, activity modification, hand therapy, corticosteroid injections, and analgesics for a few weeks [3, 4]. If symptoms progress, surgical techniques such as wrist arthroscopy are often suggested [1, 5]. Currently, there is no golden standard procedure and not one surgical procedure is superior to the other.

In previous literature, several arthroscopic methods such as inside-out, outside-in, all-inside, and all-arthroscopic methods all allow visualization, decrease in soft tissue injury, and increase in wrist motion [4, 6, 7]. Arthroscopic-assisted transosseous suture anchor repair, a commonly used procedure, has an advantage in obviating the need for an open capsulotomy with a capsular flap of the original method. This minimizes soft tissue scarring, fibrosis, and stiffness [8]. All techniques are well reported in the literature, consisting of good to excellent results that persist over time in 60% to 90% of the cases [7].

To our knowledge, only two direct comparison studies were assessing open or arthroscopic repairs of TFCC tears. One study by Anderson and their colleagues in 2008 had 75 patients total, using the visual analog pain scale (VAS) and the Mayo Modified Wrist score. They concluded that no significant differences were found in clinical outcomes after the three to four years of follow-up [9]. The other study by Chou and Lee was reported in 2001 when they compared preoperative and one-year postoperative [1]. No other comparative studies were published on the other techniques of TFCC repairs. In addition, the prevalence of TFCC cases is relatively low in Taiwan, and there was only one article published in the literature back in 2002 in 37 patients with TFCC that underwent wrist arthroscopy comparing the outcomes preoperative and postoperative was published [10]. Therefore, the purpose is 1) to compare flexion and extension range of motion (ROM), grip strength, and pain scale in all-inside arthroscopic suture anchor and arthroscopic transosseous suture technique, and 2) to understand how to manage the surgical treatments on TFCC cases in Taiwan thoroughly to see which surgical best suits the patients’ needs. Our research questions were 1) whether both techniques had significant improvements postoperative in six months after for VAS, ROM, and grip strength, and 2) whether there were any significant differences between the two techniques in VAS, ROM, and grip strength postoperative.

## Method

### Study design and patients

This study was a retrospective, comparative study at Fu Jen Catholic University Hospital (FJCUH). After the approval from the Research Ethics Committee, all surgical and medical linkage database systems were queried to obtain all patients from 2017 to 2019. Patients were included if 1) they had underwent arthroscopic surgical repair of a TFCC repair during the period 2017 to 2019 and 2) they tested positive using the fovea sign. If patients presented with 1) previous hand/wrist/elbow surgeries on the same side, 2) had persistent ulnar-sided wrist pain, and poor response from conservative treatment including bracing, physical therapy, and local injection for at least three months, and 3) had other potential causes or diagnoses in addition to TFCC, they were excluded from this study. In total, 22 from the all-inside arthroscopic suture anchor and another eight from the arthroscopic transosseous suture were included.

### All-inside arthroscopic suture anchor surgical procedure

**Fig. 1:**
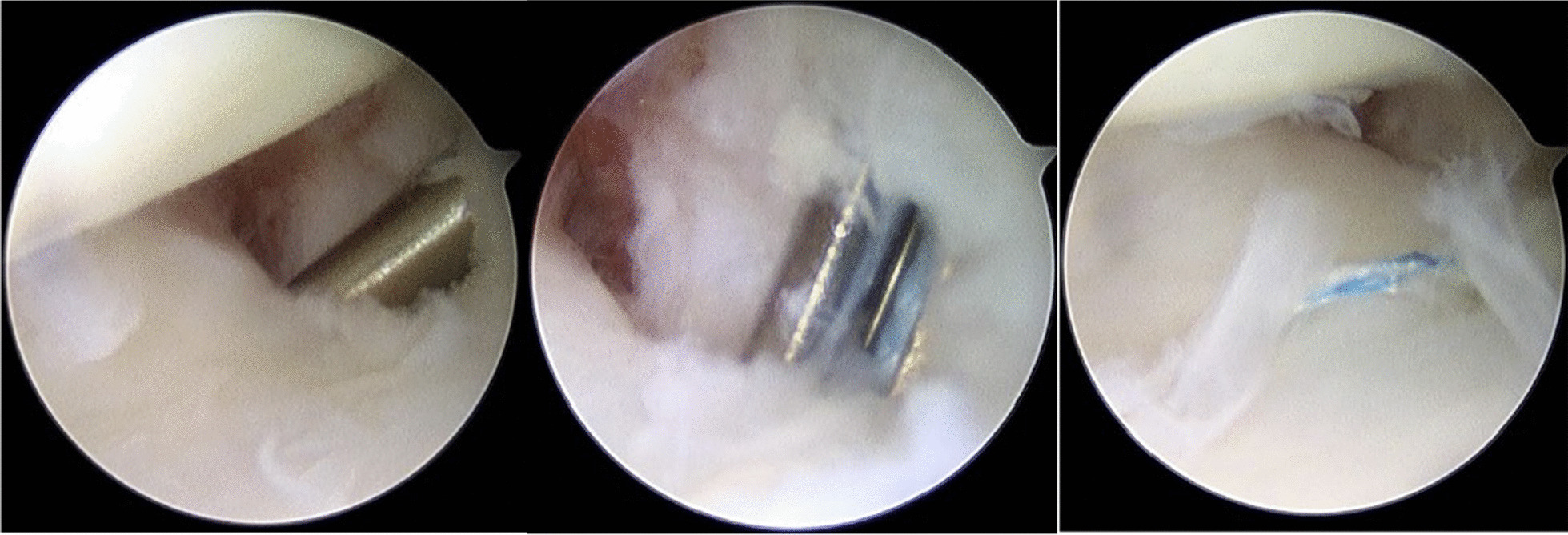
Three continuous timeframes of on-site arthroscopic camera when doing the hook test in an all-inside arthroscopic suture anchor surgical procedure

**Fig. 2:**
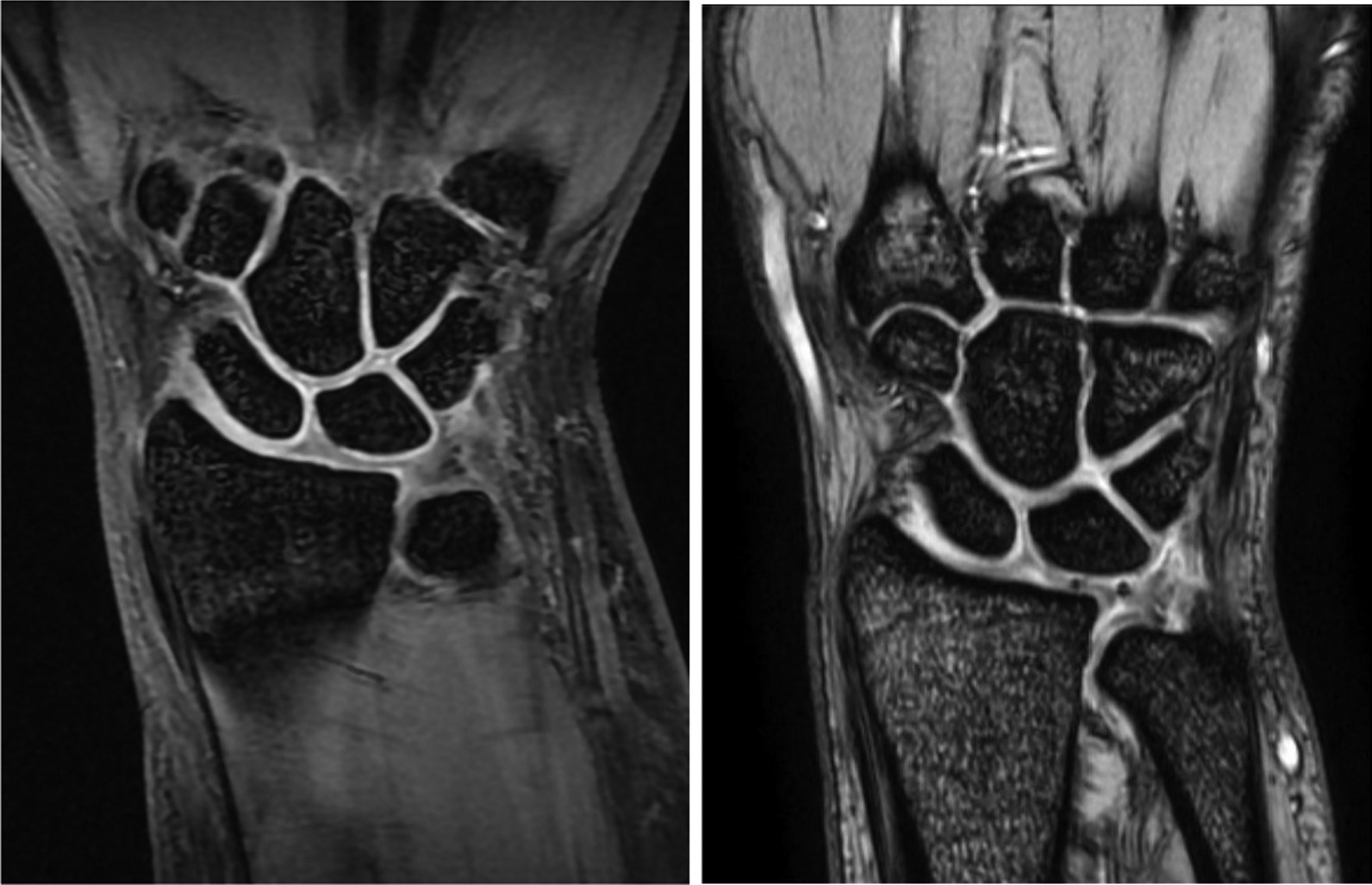
A MRI view example of a patient before (left) and after (right) their all-inside arthroscopic suture anchor surgical procedure

A hand orthopedic surgeon was in charge of all surgical procedures in all-inside arthroscopic suture anchors. This technique is a modified version from Park [6], using a 1.4-mm all-suture suture anchor and knots. The wrist was suspended with 5 kg of traction in a traction tower. With using the arthroscopic field, the hook test was performed to diagnose the location of the fovea tear of the TFCC through the 6R portal, as seen in Fig. 1. A slotted cannula combined with the obturator was pushed into the 6R portal, and the obturator was removed. After the localization of the fovea region with the identification of extensor carpi ulnaris tendon sheath, a suture anchor was inserted. A 21-gauge needle was set according to the pulp of the thumb. A No. 3-0 polydioxanone suture was inserted inside the needle as a relay. Viewed from the 3,4 portal, the surgeon was checking the appropriate position at which the needle penetrates the TFCC. Through the 6R portal cannula, the suture relay was inserted to pull out of the needle suture. The needle was pulled back to the space between the TFCC and the ulnar head. Afterward, the needle was moved aside to perform the second TFCC penetration. Another 19-gauge needle that carried the suture was inserted into the relay suture, which then the suture limb was pulled out. The horizontal mattress suture was made, using the loop beneath the TFCC and the two suture limbs in the 6R portal cannula. The suture tie was tightening to the capsule in the wrist supination position with traction released. Finally, we confirmed that there is no soft tissue in the suture way. After the surgery, the hook test was performed again to assess the integrity of the repair. The wounds were then closed. After the operation, a palmar reinforcement brace was applied to keep the forearm in a neutral position (Fig. 2).

### Transosseous suture surgical procedure

Another hand orthopedic surgeon was in charge of all surgical procedures for the transosseous suture. This technique was a modified version from Nakamura [11]. Two small holes were made from the ulnar cortex of the ulna. Sutures were then passed through the holes to repair the TFCC. Radiocarpal arthroscopy was done in 3, 4-, and 6U portals, and 1.9 arthroscope was used. When the hook test indicated positive, TFCC was repaired with two transosseous sutures. A line was drawn first near the ulnar neck along the axis of the ulnar styloid and olecranon. The retinaculum was incised and the ulnar cortex was exposed. An aiming guide was inserted into the 6R portal, and the target was aimed around the ulnar fovea region. Two tunnels of 0.045 inches in diameter were created. Under the arthroscopy guidance, a 4-O Prolene sutures were passed into the 21-gauge needle as a lasso loop and then introduced a tunnel that passed through TFCC. A 2-O TiCron suture was then passed through a 21-gauge needle. The needle was inserted distal to the ulnar styloid into the radiocarpal joint, and the suture went through the lasso loop. The Prolene suture was retrieved, and the TiCron suture was brought out of the ulnar cortex. The TiCron sutures were tightened, respectively. The wound was then sutured and the operation was completed. After the operation, a long arm splint was applied to keep the forearm in a neutral position.

### Outcome measurements

Outcome tools included the use of 1) patient’s subjective assessment of pain using a standard VAS, 2) wrist ROM, 3) grip strength test, and 4) fovea sign test. VAS was represented by either a horizontal or vertical line, 10 cm long anchored at the extremes by two verbal descriptors referring to the pain status. The patients were asked to mark a point on the line that best describes the current pain with 0 having no pain and 10 being the worst pain [12]. Wrist flexion and extension ROM were measured using a goniometer. Dynamometer (Swedlay’s Dynamo Meter, Tokyo, Japan) was used to measure grip strength, asking patients to perform three times to calculate the average. The fovea sign test was then used to see the ulnar-sided wrist pain. If they felt tenderness or pain with pressing the examiner’s thumb distally and deep into the interval between the ulnar styloid process and flexor carpi ulnaris tendon was identified, a positive sign was marked [7]. A previous study has shown a sensitivity of 95.2% and a specificity of 86.5% [13].

### Procedures

Their baseline data such as age, weight, height, body mass index (BMI), flexion and extension ROM, VAS, grip strength, and fovea sign test were collected preoperatively. Patients also went through imaging examinations on the MRI verifying the presence of TFCC. Patients were given an option to choose their appointed orthopedic surgeon to decide their scheduled time point. Both surgical procedures were proceeded with two different orthopedic surgeons to keep the consistency throughout all cases. After their successful surgery, the patients were asked to come back the following three to six weeks to see if there were any complications. If there were no complications, they were asked to continue their post-analysis comparison in their ROM, VAS, and grip strength test after six months. An additional six-month follow-up was optional if they wanted to revisit.

### Statistical analysis

SPSS Statistical software (version 20.0, SPSS Inc. Chicago, Illinois) was used to analyze all statistical data. G Power Analysis software 3.1.9.7 (Erdfelder, Faul, & Buchner, 1996) was used to input two tails, the effect size of 0.8, power (1 − *β* error probability) of 0.95, and *α* error probability of 0.05. The baseline characteristics of the participants will be reported as descriptive statistics, with continuous and categorical variables, expressed in means and standard deviation (SD). The Chi-square test was used for nonparametric statistical analysis of categorical information. Mann–Whitney U test was used for nonparametric analysis of continuous variables. Outcome measurements from preoperative and postoperative were compared using the 2-sample Student’s t test for continuous variables (grip strength, ROM, and VAS). The statistically significant difference will be defined as *p* < 0.05.

## Results

Table 1 shows the baseline characteristics of 22 patients under the all-inside arthroscopic repair and eight patients under the transosseous arthroscopic repair. Most patients were classified by the Palmer classification, with 19 patients in IB, one patient in IA, and two patients with a combination of both IB and IA. We did not perform any reoperations at any time of their follow-ups. None of the baseline characteristics showed any significant differences between each group. Table 2 displays the comparative outcomes of VAS, ROM, and grip strength in patients preoperative and six months postoperative. From the first research question, there was a significant increase in all the outcomes from preoperative to postoperative in both groups except for grip strength in the transosseous group (*p* = 0.11). Table 3 shows the clinical outcome comparison between all-inside and transosseous arthroscopic repair groups. For the second research question, none of the clinical outcomes had significant differences except for postoperative flexion ROM (*p* = 0.01).

**Table 1:** Demographic data

	All-inside (*n* = 22)	Transosseous (*n* = 8)	
Gender	14F/8M	4F/4M	
Age (years)	31.50 ± 15.09	28.38 ± 6.55	*p* = 0.44
BMI (kg/m^2^)	21.65 ± 3.22	25.45 ± 5.56	*p* = 0.10
Weight (kg)	59.27 ± 10.69	70.38 ± 20.70	*p* = 0.18
Height (cm)	165.14 ± 5.82	165 ± 9.71	*p* = 0.97

Values are represented as mean ± SD

TFCC, triangular fibrocartilage complex; BMI, body mass index

**Table 2:** Clinical outcomes of all-inside and transosseous group

	All-inside (n = 22)		Transosseous (n = 8)	
	Preoperative	Postoperative	Preoperative	Postoperative
Flexion ROM (*°*)	42.77 ± 6.71	62.77 ± 3.60 *p* < .0001*	37.87 ± 6.51	57.87 ± 4.45 *p* < .0001*
Extension ROM (*°*)	44.95 ± 7.06	61.13 ± 4.30 *p* < .0001*	39.87 ± 6.46	61 ± 2 *p* < .0001*
Grip strength (kg)	22 ± 4.97	29.91 ± 5.10 *p* < .0001*	23 ± 8.88	30.63 ± 9.41 p < .11
VAS (mm)	5.54 ± 0.86	0.91 ± 0.87 *p* < .0001*	6.0 ± 1.19	0.88 ± 0.64 *p* < .001*

Values are represented as mean ± SD

VAS, visual analogue scale; ROM, range of motion

*Significant difference

**Table 3:** Clinical outcomes comparison between all-inside and transosseous group

	All-inside vs transosseous *p* value
Pre-OP flexion ROM (*°*)	*p* = 0.09
Post-OP flexion ROM (*°*)	*p* = 0.01*
Pre-OP extension ROM (*°*)	*p* = 0.08
Post-OP extension ROM (*°*)	*p* = 0.09
Pre-OP grip strength (kg)	*p* = 0.77
Post-OP grip strength (kg)	*p* = 0.84
Pre-OP VAS (mm)	*p* = 0.34
Post-OP VAS (mm)	*p* = 0.9

Values are represented as mean ± SD

VAS, visual analogue scale; ROM, range of motion

*Significant difference

A total of 13 patients revisited the clinic in their one year after their first 6 months to do a further postoperative measurement that were additionally analyzed from the database. Eight patients who were follow-up for a year after repair reported that pain and functional improvements were better than the first six months after post-surgery. However, after analyzing their ROM and grip strength, there was no significant difference between preoperative and postoperative one year nor was there a better value of patients who had a shorter follow-up time.

## Discussion

This study design was a retrospective comparative study comparing clinical outcomes between the all-inside arthroscopic suture anchors or arthroscopic transosseous suture techniques. Different surgical repair techniques have been presented in the last few decades; however, comparative studies are rare, and a systemic review and assessment of the results have been lacking. When surgical treatment is warranted, options for surgical intervention in our hospital included both all-inside arthroscopic suture anchors and arthroscopic transosseous suture techniques. Both treatment goals were for patients with TFCC tears to have stable DRUJ with full, painless wrist motion that can return to daily life activities; however, the uncertainty of which technique was better than the other was unknown.

Most of the patients included in this study were recommended to repair surgically since most cases were Palmer Class 1B tears that often cause instability of the DRUJ. However, this study also included a combination of both Palmer Class 1A and 1B as 1A is commonly known as traumatic TFCC and was debrided when conservative treatment failed [5].

Both surgeons that were in charge of their preferred surgical procedures both underwent arthroscopic techniques. As arthroscopic technique reveals to have continuous advancement in direct visualization and magnified view, this might allow decrease injury and destruction around the tissues [14, 15]. However, previous studies mainly in Japan [16–19] have reported the importance of DRUJ arthroscopies to diagnose a rupture of the deep fibers. Radiocarpal arthroscopies that were used in this study could not visualize the foveal insertion of the deep fibers in the cases where a superficial portion of TFCC was not torn [3]. In addition, it could not identify pathological findings, such as the proximal surface of the articular disk relating to ulnar wrist pain, that DRUJ arthroscopies would find according to Yamamoto et al. [16]. That is why hook tests were used during both procedures in this study were crucial in showing good specificity and sensitivity for recognizing proximal TFCC tears [20]. The hook test confirms a high correlation with DRUJ arthroscopy, confirming that DRUJ arthroscopy is no longer required to diagnose a rupture or avulsion of proximal TFCC. In addition, the narrowness of the joint that is required discourages surgeons to practice and therefore is rarely applied in clinical settings.

The only direct comparative repair techniques from systematic reviews were open and arthroscopic repairs [9, 15, 21]. Robba et al. [15] reported a systemic review from patients that had 1B TFCC tears from 10 studies. They concluded that both techniques provided similar good outcomes with no differences. Nonetheless, most of the current evidence consists primarily of retrospective case series which demonstrates a lack of high-quality evidence to draw conclusions, suggesting the superiority over one technique or the other [15]. Another similar systemic review also drew the same conclusion after analyzing only two studies [21]. All-inside arthroscopic suture anchor technique that was used by a 2-portal technique similar to Park [6] was preferred by one of the orthopedic surgeons due to the technique being easier to perform with type IB, ID, and IIC TFCC tears, less vulnerable to ulnar nerve injury than the original Geissler technique [22], and did not require extra longitudinal incision [1, 3]. Transosseous suture technique that was modified to Nakamura [11] is suited for wrists that had an ulnar neutral or minus variance. If it is positive ulnar variance, the shear stress between the ulnar head and the suture site of the TFCC may rupture the sutures. From Nakamura’s transosseous suture technique, they revealed that 15 out of 24 patients result in no pain with a clinical outcome range from good to excellent in 79% of patients.

Different techniques and modifications are still introduced and described in many studies. In Taiwan, receiving TFCC cases is relatively small, even though the exact numbers are unknown. In our district hospital, the TFCC cases received each year are roughly only five to ten cases. Not all hospitals in Taiwan can offer these two techniques at the same place as these techniques are fairly new. To our knowledge, there is only one clinical trial analyzing TFCC patients in Taiwan that was published in 2002 [23]. A total of 37 patients were collected within three years from 1996 to 1999 using inside-out arthroscopy. Since then, newer modified procedures have been discussed in several articles; however, there are limited direct comparisons between each technique. Clinical decision making seemed to have a significant impact on choosing the types of surgical treatment for TFCC. Although the choice of techniques may be on discretion and preferences, striving for optimal function and quality of life for patients is same for all techniques.

VAS, Mayo Modified Wrist score, Disabilities of the Arm, Shoulder, and Hand score, grip strength, and ROM are usually assessed in patients with TFCC. At follow-up, DRUJ stability was restored in most of the patients. Although no direct previous studies can be compared, similar results in the increase in VAS and grip strength after arthroscopic repairs of TFCC have been reported ranging from satisfactory to excellent [24–26]. From our research question, a direct comparison between the two repairs did not have any significant differences except for postoperative flexion ROM; however, there were several increase in improvements for preoperative and postoperative in both of the repairs. Our study follow-up time was 6 months. A longer follow-up time may allow a further increase in improvements in pain, symptoms, and function [26]. With the additional 13 patients that revisited the clinic one year after to do a further postoperative measurement, we could not detect any significant differences when compared to their six-month visit as their improvements in their wrist were roughly the same.

This study has limitations that could take into consideration in future studies using a long-term prospective randomized study design. The total cases from then until now has been two years; however, the prevalence of TFCC cases in Taiwan is relatively low. The relatively small sample size from the low incidence of surgical procedures performed for TFCC repairs and a short follow-up period of 6 months were inevitable. Most patients were hard to follow up even further due to the difficulty of finding time back in the hospital. In addition, the two techniques were performed by two different surgeons, due to their preferred choice of that procedure in addition to the rarity of an orthopedic surgeon that can do two different TFCC procedures. However, after knowing the time needed to accumulate an adequate larger sample size as well as the importance of analyzing the clinical correlation between TFCC and DRUJ pathology, clinical comparison between the two techniques will continue to practice into clinical trial soon with longer follow-up times with subgrouping into TFCC classifications. Both surgical techniques are appropriate solutions to treat patients with TFCC.

## Data Availability

Currently, no public links are available to these information datasets. These data will be made available to others after appropriate data privacy and human subject approvals needed by the institution. Requests are welcomed and are to be sent to Yjchen71@gmail.com.
